# Dietary niacin intake and mortality among chronic kidney disease patients

**DOI:** 10.3389/fnut.2024.1435297

**Published:** 2024-11-21

**Authors:** Zhengxi Zhou, Xiaotian Yao

**Affiliations:** ^1^Department of Urology, Ningbo Mingzhou Hospital, Zhejiang, China; ^2^The Division of Nephrology, The Sixth Affiliated Hospital, Sun Yat-sen University, Guangzhou, Guangdong, China; ^3^Biomedical Innovation Center, The Sixth Affiliated Hospital, Sun Yat-sen University, Guangzhou, Guangdong, China

**Keywords:** niacin, CKD, NHANES, all-cause mortality, cardiac mortality

## Abstract

**Background:**

The relationship between niacin and the risk of mortality in chronic kidney disease (CKD) patients remains unclear. This study aims to investigate the potential correlation.

**Methods:**

This cohort study utilized data from the 2003–2018 National Health and Nutrition Examination Survey (NHANES). The study included 6,110 patients with CKD aged 18 years or older. Weighted Cox proportional hazards models and restricted cubic splines (RCS) were employed to estimate hazard ratios for all-cause mortality and cardiovascular disease (CVD) mortality. Niacin intake was estimated using the 24 h dietary recall method, based on the type and amount of food consumed. All-cause mortality and cardiac mortality rates were determined using National Death Index (NDI) mortality data (as of 31 December 2018).

**Results:**

The median niacin intake was 20.89 mg/day, with an interquartile range of 15.67–27.99 mg/day. During the follow-up period (median of 87 months), there were 1,984 all-cause deaths, including 714 CVD deaths. Compared with low niacin intake, the multivariate-adjusted hazard ratio for dietary intake of 22 mg or higher was 0.71 (95% CI, 0.57–0.88) for all-cause mortality and 0.75 (95% CI, 0.57, 0.98) for CVD mortality.

**Conclusion:**

Dietary niacin intake is associated with a reduction in all-cause and cardiac mortality among CKD patients.

## Introduction

Chronic kidney disease (CKD) is an important factor in non-communicable disease morbidity and mortality, representing both a global public health problem and a large medical and financial burden (1, 2). Globally, the global number of deaths from CKD is significant and increasing, with the incidence of CKD increasing by 89%, its prevalence by 87%, and deaths caused by CKD (3) in CKD patients. Therefore, reducing the disease burden and the risk of death from CKD is particularly important.

The importance of nutrition in promoting health has garnered significant attention. Niacin (vitamin B3) serves as a precursor for the synthesis of nicotinamide adenine dinucleotide phosphate (NADP) and nicotinamide adenine dinucleotide (NAD), both of which are crucial in mitigating oxidative stress, inflammation, and endothelial dysfunction (4) These properties have positioned niacin as a significant target of research aimed at reducing all-cause and cardiovascular mortality in CKD patients. Niacin Lower low-density lipoprotein (LDL) and triacylglycerol levels, while increasing high-density lipoprotein (HDL) levels, which helps to improve cardiovascular health (5). In most prospective studies, triglyceride and / or high-density lipoprotein cholesterol (HDL-C) levels were identified as predictors of the development or progression of CKD (6). Oxidative stress has been identified as a major factor in the progression of CKD (7). Niacin may attenuate systemic inflammation and lower the risk of cardiovascular events by inhibiting the production of pro-inflammatory cytokines (6). Furthermore, its protective effects on endothelial cells may enhance vasodilatory function, thereby contributing to a further reduction in cardiovascular risk (8–10).

However, there are still no prospective studies evaluating the association between intake of niacin from food and mortality risk in patients with CKD. This is the first study to investigate the relationship between dietary intake of niacin and all-cause mortality and CVD mortality in CKD patients, using a nationally representative sample of adults in the United States.

## Methods

### Study design and demographics

The National Health and Nutrition Examination Survey (NHANES) is a cross-sectional study conducted by the Centers for Disease Control and Prevention (CDC) to assess the health and nutritional status of adults and children in the United States. This cohort study used data from the NHANES, a screening program to assess the health and nutritional status of non-institutional general U. S. residents. NHANES adopts a multi-stage probability sampling design, and over-sampling, stratification and weighting to ensure its data accurately represent the American population, with information collected every 2 years (11). We used NHANES adult data for 8 cycles from 2003 to 2018. Of the 80,312 people from 2003 to 2018, 7,983 people had CKD. Participants with missing baseline creatinine, urinary protein, survival data, baseline niacin data will be further excluded.

### Determination of dietary niacin intake

The dietary interview was conducted in collaboration with the United States Department of Agriculture (USDA) and the US Department of Health and Human Services, utilizing a standardized automated multiple-pass method (AMPM). Nutrient and food composition data for all foods were calculated using the USDA Diet Research Food and Nutrition Database. Dietary niacin intake was assessed through a 24 h dietary recall interview, where participants reported the type and amount of food consumed in the 24 h preceding the interview. The first food recall was administered in person at the Mobile Inspection Center, while the second recall took place via telephone interviews approximately 3 to 10 days later. For this study, the average of the participants’ two dietary recalls was used.

### Definition of CKD

CKD encompasses chronic renal structure and dysfunction arising from various causes, characterized by a history of renal impairment lasting more than 3 months. This includes normal and abnormal renal GFR pathology, abnormal blood or urine composition, abnormal imaging findings, or an unexplained decline in GFR (less than 60 mL/min/1.73m^2^) persisting for more than 3 months (12). The eGFR is calculated using the formula from the 2009 Chronic Kidney Disease Epidemiology Collaboration (CKD-EPI) (13).

### Ascertainment of mortality

The study accessed the NDI through December 31, 2019, to gather data on deaths and follow-up durations. Causes of death were identified using codes from the International Statistical Classification of Diseases, Tenth Revision (ICD-10). CVD mortality encompasses various causes of death. Cardiovascular mortality was specifically defined as deaths from cerebrovascular disease (codes I60-I69) and heart disease (codes I00-I09, I11, I13, and I20-I51). The follow-up duration was measured in months, from the date of the family interview to either the date of death or the end of the follow-up period.

### Definition of covariates

Participant demographic information was collected through a family interview questionnaire. Age was grouped into two categories: under 60 years and 60 years or older. Ethnicity was divided into five categories: White, Black, Mexican American, and Other. Household income was categorized into three levels based on the poverty index: above 3.5 (high income), between 1.3 and 3.5 (middle income), and below 1.3 (low income). Marital status was classified as either coupled (married or in a committed relationship) or single (not married and not in a committed relationship). Educational attainment was sorted into four levels: college graduate or higher, some college or associate degree, high school graduate, and less than high school. Body mass index (BMI) was categorized as obesity (30 or above) and non-obesity (below 30). Alcohol consumption was classified as drinkers and non-drinkers, while smoking status was categorized into former smokers, current smokers, and never smokers. During physical examinations, key biometric measurements such as blood pressure, height, and BMI were taken, with BMI calculated by dividing weight in kilograms by height in meters squared. The study also included laboratory tests measuring serum urea, creatinine, carbon dioxide, uric acid, urine albumin-to-creatinine ratio (uACR), among others. Conditions like hypertension, diabetes, and CVD were diagnosed by doctors and additionally reported by the participants.

### Statistical analysis

This study utilized the complex sampling structure of NHANES to ensure that the results accurately represent the U.S. population, incorporating elements such as sample weighting, clustering, and stratification in all analyses. The baseline characteristics of participants with CKD were detailed using statistical techniques. For continuous variables, we presented weighted medians, interquartile ranges (IQRs), and their respective confidence intervals (CIs). Categorical variables were described through weighted percentages. For comparing continuous variables, multiple sample tests were employed, while categorical variables were analyzed using Fisher’s exact test or the Chi-squared test.

To address missing data, the study implemented a sophisticated multilevel imputation technique tailored for survey data, utilizing the “jomo” R package (14). Ten imputed datasets were generated using a Gibbs sampling method, starting with a 500-iteration burn-in phase followed by 100 updates. This approach ensured that each imputed dataset was statistically independent. For the statistical analysis, a complete case approach was used. “jomo” R package is particularly advantageous due to its flexibility in handling both continuous and categorical variables, as well as its ability to model complex relationships within multivariate datasets. This method allows for capturing the uncertainty associated with imputed values, thereby enhancing the robustness of our estimates. The optimal niacin cutoff, which showed the strongest correlation with survival outcomes, was identified using the MSRSM provided by the “maxstat” package. Based on this cutoff, participants were divided into groups with high and low niacin levels (15).

We selected confounding variables including basic demographic information, laboratory information related to CKD, niacin-related lipid indicators, diet-related indicators, and medication. RCS regression was utilized to investigate potential non-linear relationships between niacin intake and mortality risk among CKD patients. We evaluated the existence of non-linearity by conducting the likelihood ratio test. To further examine the association between niacin and mortality risk, survey-weighted univariate and multivariate Cox regression analyses were conducted (weights: “wtdr2d”). The proportional hazards assumption was tested using Schoenfeld residuals, and no violation was observed. Five models were constructed. Crude Model did not adjust for any covariate. Model 1 adjusted for age, gender, marital, poverty, ethnicity, smoking, drinking, and obesity. Model 2 additionally adjusted for HbA1c, hemoglobin (Hb), NLR, serum uric_acid, blood_urea_nitrogen, serum bicarbonate, serum creatinine, serum albumin, and uACR. Model 3 additionally adjusted for systemic immune inflammation Index (SII), dietary energy, dietary protein, dietary_fiber, dietary sodium, dietary potassium. Model 4 additionally adjusted for CVD history, hypertension, hyperlipidemia, the use of hyperlipidemic drug, the use of hypertensive drug. Potential confounding factors were considered in stratified analysis, taking into account factors such as age, serum creatinine, uACR, BMI, uric acid levels, blood urea nitrogen levels, bicarbonate levels, Hb, serum albumin, HbA1c, and etc. The significance of the interaction between each stratification factor was assessed, with *p*-values presented accordingly. All statistical analyses were conducted using R version 4.3.1 (R Foundation for Statistical Computing, Vienna, Austria). A *p*-value of less than 0.05 was deemed statistically significant in all two-tailed tests.

## Results

**Figure 1:**
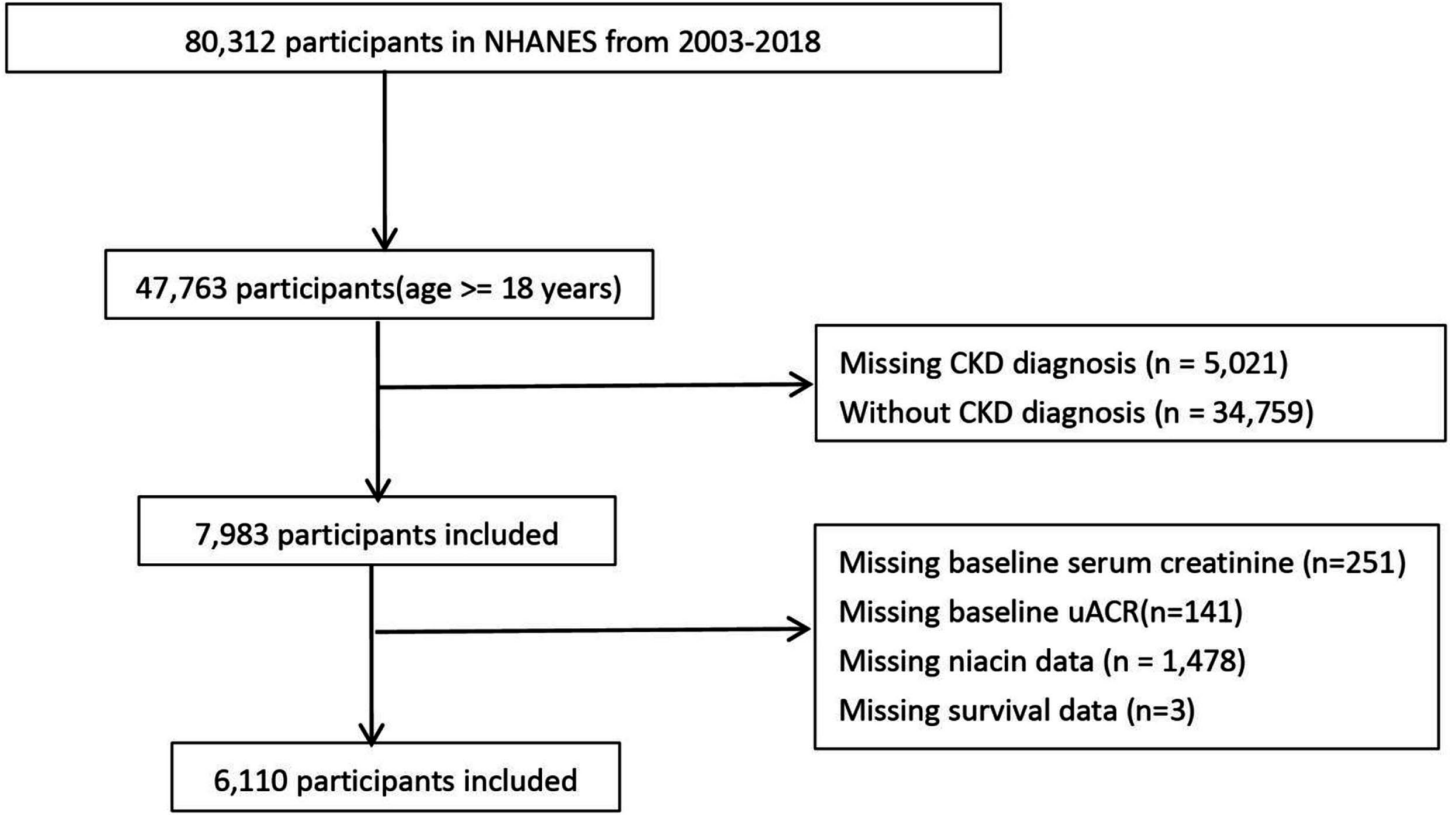
Flowchart of study population.

**Figure 2:**
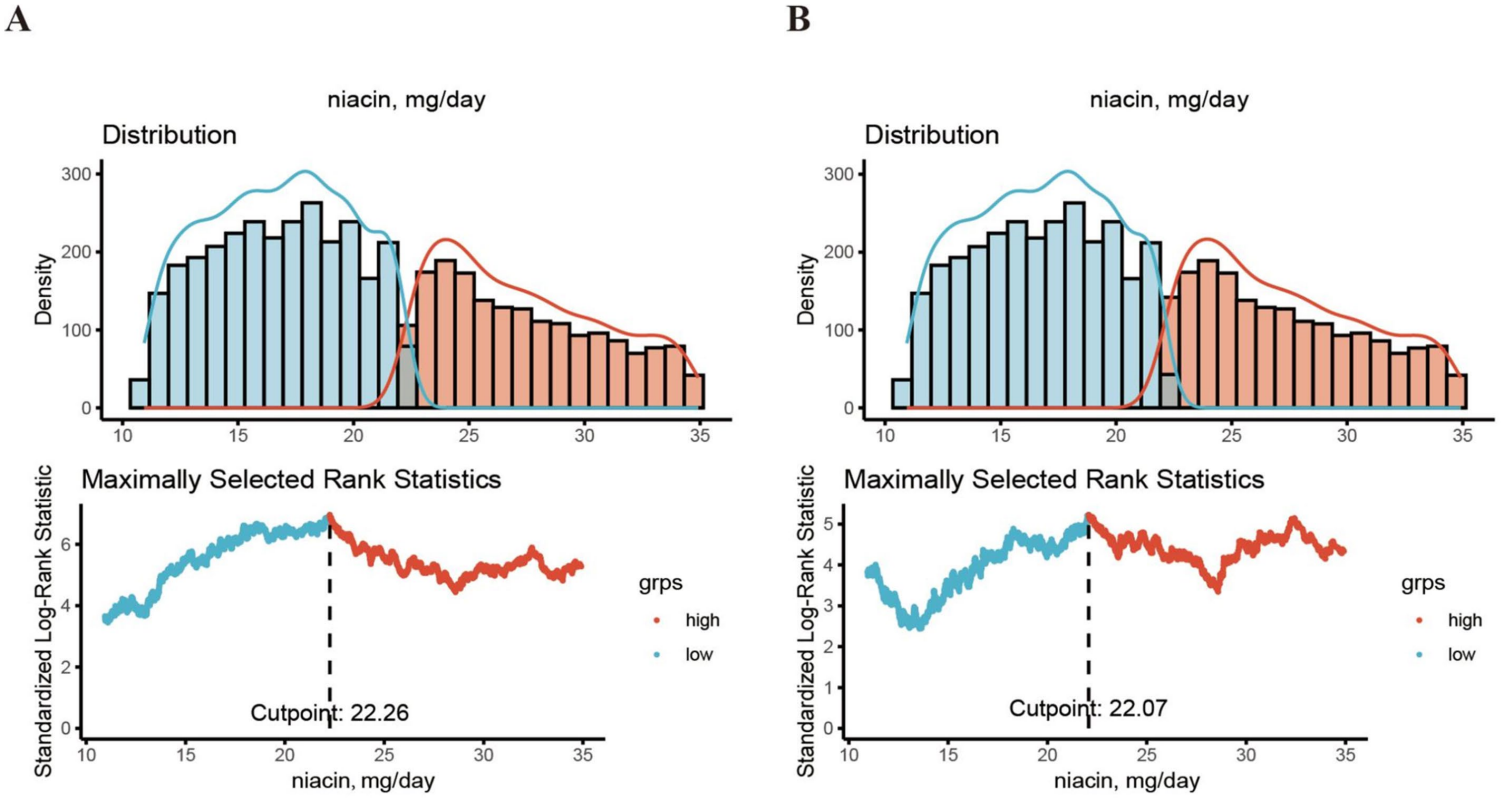
The cutoff point was calculated using the MMRSM using maxstat package. The cutoff point was calculated using the MMRSM for all-cause mortality **(A)** and CVD mortality **(B)**.

A total of 6,110 CKD patients were included in this study (Figure 1), with weighted mean age 59.93 years, 2,835 males (weighted 42.48%). The cohort were grouped into high level and low level according to the optimal niacin cutoff (22.26 for CVD mortality; 22.07 for CVD mortality). The optimal NLR cutoff was calculated by the MSRSM and corresponded the most significant correlation for survival (Figure 2). Participants exhibiting higher niacin intake levels were more inclined to be younger, male, without hypertension, without cardiovascular history, more energy intake, more protein intake, higher uric acid, and more urinary protein (Table 1). There was no significance between serum creatinine, blood urea nitrogen, serum bicarbonate and SII in different level.

**Table 1:** Baseline characteristics of the study population.

Characteristic	Total	Niacin, mg/day		*p*-value
		High	Low	
		(22.26–117.43)	(0–22.26)	
Age	59.93 (59.27, 60.59)	57.21 (56.11, 58.31)	62.10 (61.20, 63.00)	<0.0001
Age strata				<0.0001
<60	42.16 (39.01, 45.32)	48.88 (45.82, 51.94)	36.81 (34.19, 39.43)	
> = 60	57.84 (54.25, 61.42)	51.12 (48.06, 54.18)	63.19 (60.57, 65.81)	
Gender				<0.0001
Female	57.52 (53.41, 61.63)	41.11 (38.03, 44.20)	70.59 (68.45, 72.74)	
Male	42.48 (39.62, 45.33)	58.89 (55.80, 61.97)	29.41 (27.26, 31.55)	
BMI, kg/m^2^	30.13 (29.80, 30.46)	30.54 (30.05, 31.03)	29.80 (29.42, 30.18)	0.01
SBP, mmHg	131.31 (130.40, 132.21)	129.31 (128.07, 130.55)	132.90 (131.71, 134.08)	<0.0001
DBP, mmHg	69.49 (68.93, 70.05)	70.93 (70.15, 71.71)	68.35 (67.72, 68.97)	<0.0001
Hypertension				0.01
No	33.95 (31.04, 36.87)	36.91 (33.75, 40.06)	31.60 (29.33, 33.88)	
Yes	66.05 (62.07, 70.02)	63.09 (59.94, 66.25)	68.40 (66.12, 70.67)	
DM				0.26
No	58.62 (54.57, 62.66)	59.23 (55.98, 62.49)	58.13 (55.67, 60.59)	
DM	33.16 (30.69, 35.64)	31.74 (28.84, 34.64)	34.30 (31.87, 36.73)	
IFG	4.49 (3.69, 5.29)	5.26 (3.96, 6.56)	3.88 (3.04, 4.72)	
IGT	3.73 (3.00, 4.46)	3.77 (2.43, 5.11)	3.70 (2.97, 4.43)	
CVD history				<0.001
No	75.21 (70.73, 79.69)	78.17 (75.97, 80.37)	72.85 (70.70, 75.00)	
Yes	24.79 (22.67, 26.91)	21.83 (19.63, 24.03)	27.15 (25.00, 29.30)	
Education				<0.0001
<High school graduate	21.98 (19.91, 24.06)	16.28 (14.32, 18.25)	26.52 (23.92, 29.13)	
High school graduate	26.39 (23.92, 28.87)	24.73 (21.91, 27.55)	27.72 (25.58, 29.86)	
Some college or associates degree	29.50 (26.98, 32.03)	32.84 (29.79, 35.89)	26.85 (24.54, 29.15)	
College graduate or above	22.12 (19.38, 24.85)	26.14 (22.76, 29.52)	18.91 (16.63, 21.20)	
Ethnicity				0.24
White	71.13 (65.15, 77.12)	71.17 (67.77, 74.57)	71.11 (68.32, 73.89)	
Black	11.45 (10.07, 12.83)	10.47 (8.69, 12.25)	12.24 (10.45, 14.02)	
Mexican American	7.16 (5.94, 8.38)	7.35 (5.70, 9.01)	7.00 (5.64, 8.36)	
Other	10.26 (9.06, 11.45)	11.01 (8.96, 13.05)	9.66 (8.24, 11.07)	
Marital				<0.0001
Single	41.11 (38.23, 43.99)	35.51 (32.25, 38.77)	45.57 (43.03, 48.11)	
Couple	58.89 (54.54, 63.24)	64.49 (61.23, 67.75)	54.43 (51.89, 56.97)	
Poverty				<0.0001
<1.3	24.47 (22.60, 26.35)	21.11 (18.45, 23.77)	27.15 (25.02, 29.29)	
1.3–3.5	41.70 (38.86, 44.55)	39.67 (37.09, 42.25)	43.32 (40.98, 45.66)	
>3.5	33.82 (30.43, 37.22)	39.22 (35.57, 42.87)	29.53 (26.77, 32.29)	
Obesity				0.12
No	55.15 (51.43, 58.86)	53.41 (50.14, 56.67)	56.53 (54.12, 58.95)	
Yes	44.85 (41.61, 48.09)	46.59 (43.33, 49.86)	43.47 (41.05, 45.88)	
Smoking				0.08
Never	32.69 (30.18, 35.21)	34.88 (31.67, 38.09)	30.95 (28.82, 33.09)	
Former	50.58 (46.90, 54.25)	48.15 (44.87, 51.44)	52.50 (50.09, 54.92)	
Now	16.73 (14.92, 18.54)	16.97 (14.86, 19.08)	16.54 (14.30, 18.79)	
Drinking				<0.0001
No	42.58 (39.64, 45.51)	34.44 (30.98, 37.90)	49.05 (46.17, 51.94)	
Yes	57.42 (53.25, 61.60)	65.56 (62.10, 69.02)	50.95 (48.06, 53.83)	
SII	523.43 (370.00, 751.06)	517.65 (359.06, 763.73)	531.00 (380.53, 742.50)	0.36
Serum creatinine, umol/L	97.33 (95.39, 99.27)	96.51 (93.59, 99.44)	97.98 (95.55, 100.41)	0.43
eGFR				<0.0001
<=G3a	86.09 (80.98, 91.20)	90.17 (88.89, 91.45)	82.84 (81.18, 84.50)	
> = G3b	13.91 (12.68, 15.14)	9.83 (8.55, 11.11)	17.16 (15.50, 18.82)	
Serum uric acid, umol/L	354.20 (350.27, 358.12)	361.31 (355.37, 367.26)	348.53 (344.12, 352.95)	<0.001
Blood urea nitrogen, mmol/L	6.26 (6.16, 6.37)	6.20 (6.04, 6.36)	6.31 (6.17, 6.45)	0.31
Serum bicarbonate, mmol/L	25.02 (24.89, 25.14)	25.12 (24.96, 25.29)	24.93 (24.78, 25.08)	0.06
Serum triglycerides, mmol/L	1.97 (1.90, 2.04)	2.04 (1.93, 2.15)	1.91 (1.83, 1.99)	0.05
Serum total cholesterol, mmol/L	5.05 (5.00, 5.10)	5.00 (4.93, 5.07)	5.08 (5.02, 5.14)	0.09
Serum hdl cholesterol, mmol/L	1.36 (1.34, 1.38)	1.33 (1.30, 1.36)	1.39 (1.37, 1.41)	<0.001
Lymphocyte	2.16 (2.06, 2.26)	2.25 (2.05, 2.45)	2.09 (2.02, 2.16)	0.13
Neutrophil	4.63 (4.57, 4.70)	4.63 (4.53, 4.73)	4.63 (4.55, 4.72)	0.96
Hb, g/dL	13.86 (13.78, 13.93)	14.24 (14.15, 14.34)	13.55 (13.46, 13.64)	<0.0001
Albumin, g/L	41.58 (41.41, 41.76)	41.89 (41.66, 42.12)	41.34 (41.13, 41.55)	<0.0001
HbA1c	6.09 (6.04, 6.15)	6.10 (6.02, 6.19)	6.09 (6.03, 6.15)	0.81
HbA1c strata				0.47
HbA1c < 7	85.18 (80.15, 90.21)	84.63 (82.63, 86.63)	85.61 (83.77, 87.46)	
HbA1c > =7	14.82 (13.24, 16.41)	15.37 (13.37, 17.37)	14.39 (12.54, 16.23)	
uACR, mg/g	41.17 (12.56, 92.38)	43.85 (14.89, 98.41)	39.29 (11.78, 87.05)	0.03
				0.01
A1	33.99 (30.95, 37.02)	30.91 (28.16, 33.67)	36.43 (33.86, 39.00)	
A2	56.09 (52.63, 59.55)	58.40 (55.27, 61.53)	54.26 (51.71, 56.80)	
A3	9.92 (8.74, 11.10)	10.69 (8.70, 12.67)	9.31 (8.08, 10.54)	
Dietary fiber, g/day	15.78 (15.43, 16.13)	19.49 (18.93, 20.05)	12.83 (12.49, 13.16)	<0.0001
Dietary sodium, mg/day	3147.84 (3095.40, 3200.28)	3956.44 (3877.67, 4035.22)	2503.98 (2457.35, 2550.61)	<0.0001
Dietary potassium, mg	2524.92 (2484.31, 2565.54)	3136.34 (3080.19, 3192.49)	2038.08 (2000.22, 2075.93)	<0.0001
Dietary protein, g/day	74.30 (73.11, 75.48)	96.86 (95.20, 98.53)	56.33 (55.40, 57.25)	<0.0001
Dietary energy, kcal/day	1889.37 (1860.65, 1918.09)	2360.43 (2315.31, 2405.55)	1514.28 (1489.94, 1538.63)	<0.0001
				<0.0001
<1,500	32.38 (29.85, 34.91)	8.54 (6.79, 10.29)	51.37 (48.70, 54.04)	
> = 1,500	67.62 (63.45, 71.79)	91.46 (89.71, 93.21)	48.63 (45.96, 51.30)	
Hyperlipidemia				0.58
No	64.01 (60.18, 67.84)	63.44 (60.25, 66.64)	64.46 (62.25, 66.67)	
Yes	35.99 (32.92, 39.06)	36.56 (33.36, 39.75)	35.54 (33.33, 37.75)	
Hyperlipidemic drug				0.46
No	64.14 (59.91, 68.37)	64.90 (62.12, 67.67)	63.54 (60.96, 66.11)	
Yes	35.86 (33.11, 38.61)	35.10 (32.33, 37.88)	36.46 (33.89, 39.04)	
Hypertensive drug				0.002
No	42.32 (38.99, 45.64)	46.27 (43.01, 49.53)	39.17 (36.36, 41.99)	
Yes	57.68 (53.98, 61.39)	53.73 (50.47, 56.99)	60.83 (58.01, 63.64)	
Follow-up time, month	87.06 (84.52, 89.60)	89.32 (85.74, 92.90)	85.26 (82.25, 88.28)	0.06

Data were presented as weighted percentages or means (95% CI). SBP, systolic blood pressure; DBP, diastolic blood pressure; BMI, body mass index; Hb, hemoglobin; uACR, urine albumin creatine ratio; A1, <30 mg/g; A2 30–300 mg/g; A3 > 300 mg/g. eGFR, estimated glomerular filtration rate; <=G3a, eGFR > =45 mL/(min • 1.73 m^2^), > = G3b, eGFR > 45 mL/(min • 1.73 m^2^). DM, diabetes mellitus; IGT, impaired glucose tolerance; IFG, impaired fasting glucose.

### Dietary niacin intake and CVD and all-cause mortality

During the end of the follow-up, the median follow-up time was 87.06 (IQR, 84.52–89.60) months, with 1,984 CVD deaths (714 CVD deaths). In crude model, the HR for CVD mortality was 0.65 (95% CI, 0.52–0.8) in higher niacin when compared to the lower group (*p* < 0.001) (Table 2). For each unit increase of niacin (ln-transformed), the HR for CVD mortality in crude model was 0.62 (95% CI, 0.52–0.74). For CVD mortality, when compared to the lower group in model 1, model 2, model 3 and model 4, the HR in the higher niacin group was 0.8, 0.8, 0.79, 0.8, separately. In models 4, higher niacin has protective significance, but it does not reach statistical significance compared to lower niacin. For each unit increase of niacin (ln-transformed), the HRs for CVD mortality in model 1, model 2, model 3 and model 4 were 0.83, 0.79, 0.71, and 0.75, respectively. For all-cause mortality, the HR was 0.67 (95% CI, 0.58–0.77) in higher niacin when compared to the lower group (*p* < 0.001) in crude model (Table 2). For each unit increase of niacin (ln-transformed), the HR for all-cause mortality in crude model was 0.63 (95% CI, 0.54–0.73). For all-cause mortality, when compared to the lower group in model 1, model 2, model 3 and model 4, the HR in the higher niacin group were 0.8 (95% CI, 0.71–0.91), 0.83 (95% CI, 0.73–0.93), 0.8 (95% CI, 0.66–0.97), 0.81 (95% CI, 0.66–0.93), respectively (all *p* < 0.05) (Table 2). For each unit increase of niacin (ln-transformed), the HRs for all-cause mortality in model 1, model 2, model 3, and model 4 were 0.81 (95% CI, 0.69–0.95), 0.8 (95% CI, 0.68–0.95), 0.69 (95% CI, 0.55–0.86), and 0.71 (95% CI, 0.57–0.88), respectively (all *p* < 0.05) (Table 2).

**Table 2:** HR (95% CI) for all-cause and cardiac mortality based on niacin among CKD patients.

Characteristic			Niacin	Per-unit	*p*
		Low level	High level	Increment	
All-cause mortality					
	Crude model	Ref	0.67 (0.58, 0.77)	0.63 (0.54, 0.73)	<0.0001
	Model 1	Ref	0.8 (0.71, 0.91)	0.81 (0.69, 0.95)	0.01
	Model 2	Ref	0.83 (0.73, 0.93)	0.8 (0.68, 0.95)	0.01
	Model 3	Ref	0.8 (0.66, 0.97)	0.69 (0.55, 0.86)	0.001
	Model 4	Ref	0.81 (0.66, 0.93)	0.71 (0.57, 0.88)	0.002
Cardiac mortality					
	Crude model	Ref	0.65 (0.52, 0.80)	0.62 (0.52, 0.74)	<0.0001
	Model 1	Ref	0.8 (0.66, 0.97)	0.83 (0.69, 1)	0.05
	Model 2	Ref	0.8 (0.66, 0.97)	0.79 (0.66, 0.96)	0.02
	Model 3	Ref	0.79 (0.62, 1)	0.71 (0.52, 0.98)	0.03
	Model 4	Ref	0.8 (0.62, 1.02)	0.75 (0.57, 0.98)	0.04

HR (95% CI) was estimated by Cox proportional hazards model and accounted for the sample weights. Cardiac mortality was defined as I00-I09, I11, I13, I20-I51 according to the ICD-10 criteria. Niacin(ln-transformed). Crude was unadjusted. Model 1 was adjusted for age, gender, ethnicity, marital, poverty, education, smoking, drinking, obesity. Model 2 was additionally adjusted for HbA1c, Hb, NLR, uric acid, blood urea nitrogen, bicarbonate, creatinine, uACR (ln-transformed), albumin, hdl cholesterol. Model 3 was additionally adjusted for SII(ln-transformed), dietary energy kcal, dietary protein, dietary fiber intake, dietary sodium, dietary potassium. Model 4 was additionally adjusted for CVD history, hypertension, hyperlipidemia, the use of hyperlipidemic drug, the use of hypertensive drug.

**Figure 3:**
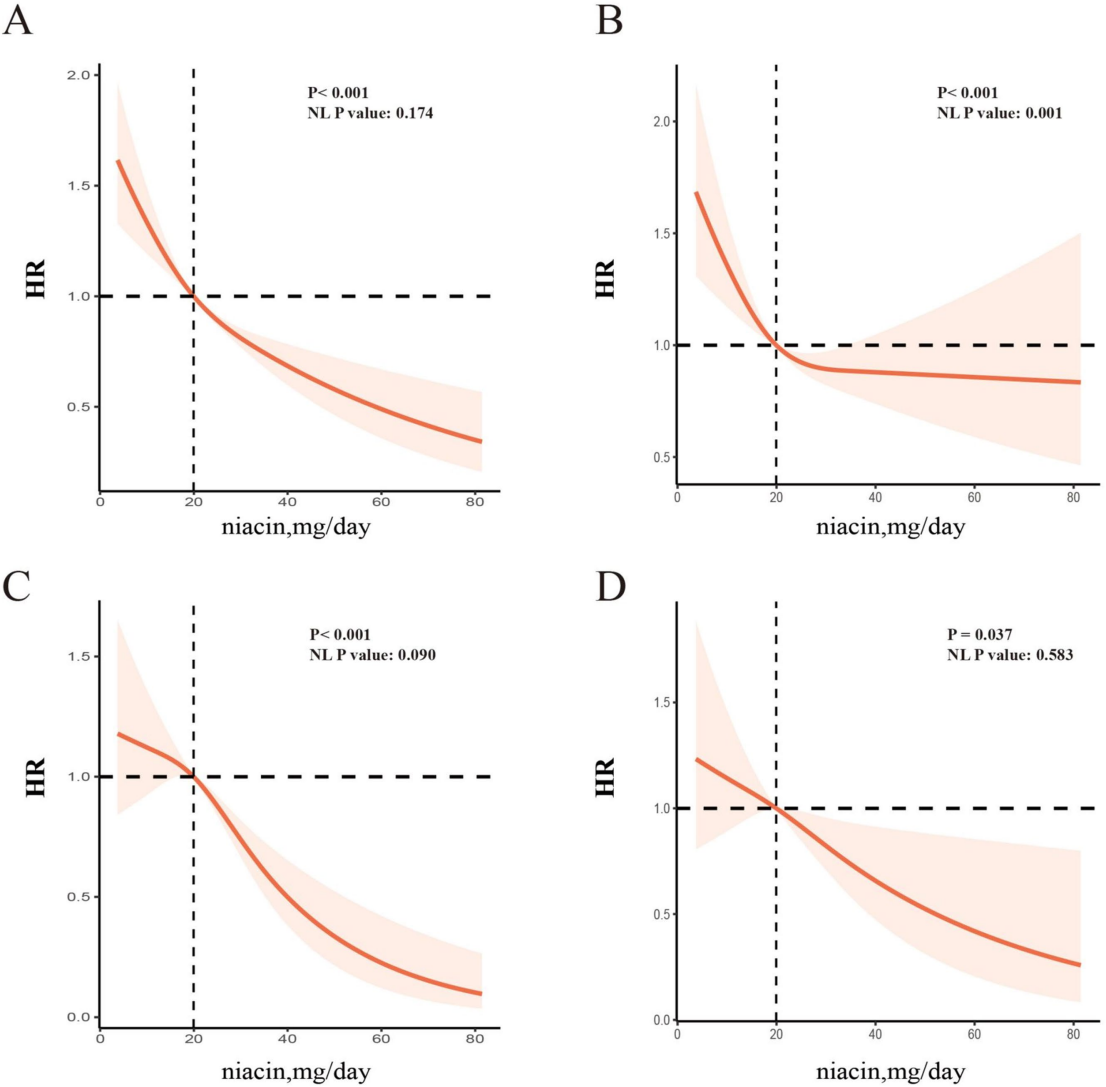
RCS analysis between niacin and all-cause mortality and cardiac mortality among CKD patients [All-cause mortality, unadjusted HR **(A)**; adjusted HR **(B)**; CVD mortality, unadjusted HR **(C)**; adjusted HR **(D)**]. The spline model was adjusted for consistent confounding factors, including age, gender, ethnicity, marital, poverty, education, smoking, drinking, obesity, HbA1c, Hb, NLR, uric acid, blood urea nitrogen, bicarbonate, creatinine, uACR (ln-transformed), albumin, hdl cholesterol, SII(ln-transformed), dietary energy kcal, dietary protein, dietary fiber intake, dietary sodium, dietary potassium, CVD history, hypertension, hyperlipidemia, the use of hyperlipidemic drug, the use of hypertensive drug. NL-P, p for non-linearity.

**Figure 4:**
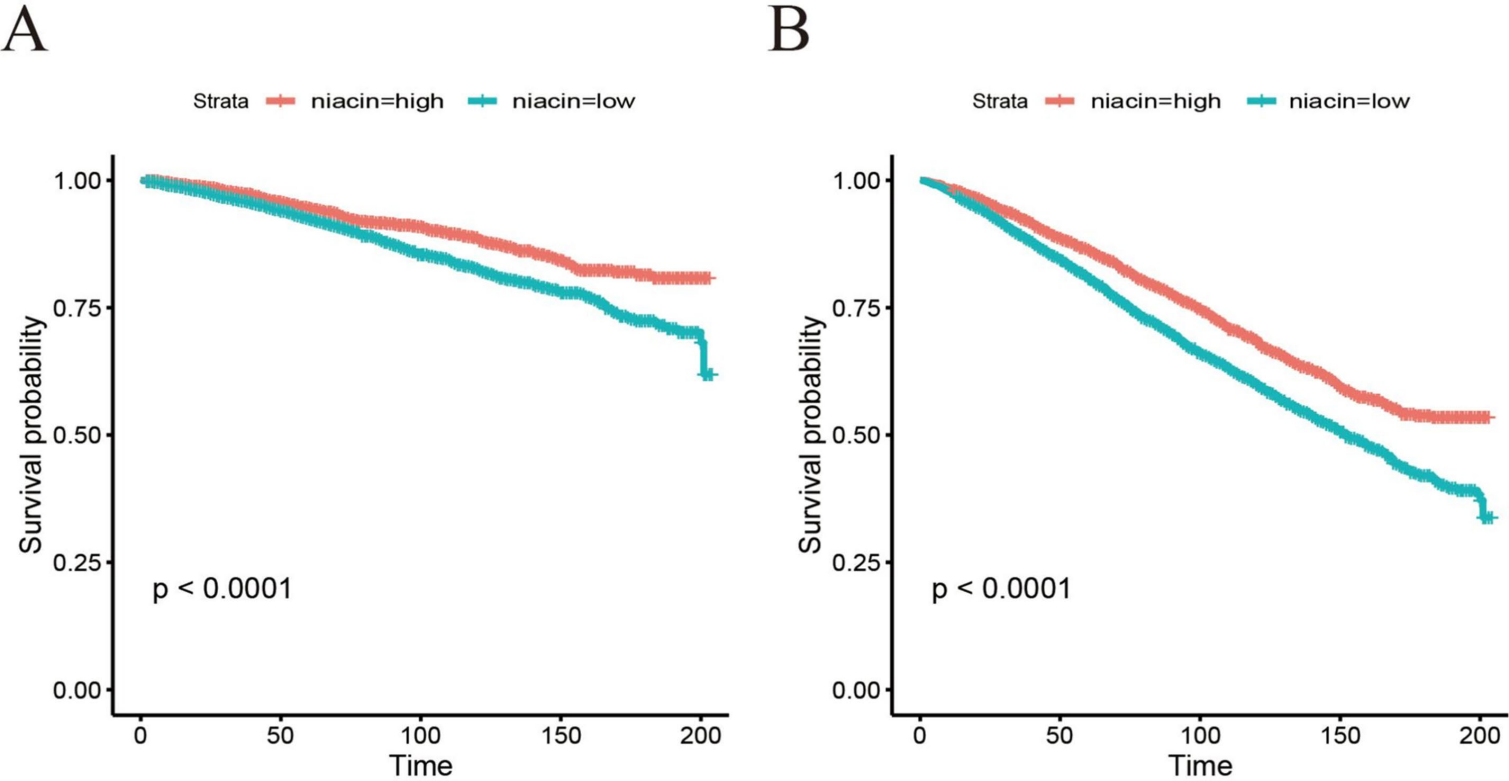
Kaplan–Meier survival curve of all-cause mortality **(A)** and CVD mortality **(B)** based on cut-off of niacin among CKD patients.

The dose–response relationship between dietary intake of niacin and CVD and all-cause mortality is shown in Figure 3. In the restricted cubic spline, there was no significant nonlinear association between dietary niacin intake and all-cause mortality (nonlinear *p* = 0.17) or CVD mortality (nonlinear *p* = 0.09) in crude model (all *p* < 0.05). After adjusting variables, the linear association changed between dietary intake of niacin and all-cause mortality (nonlinear *p* < 0.05). After adjusting the variables, there was reach the significant association between dietary niacin intake and all-cause/CVD mortality in the restricted cubic spline. In K-M plot, the high niacin level had longer survival from all-cause and CVD mortality (Figure 4).

### Stratified and sensitivity analyses

**Figure 5:**
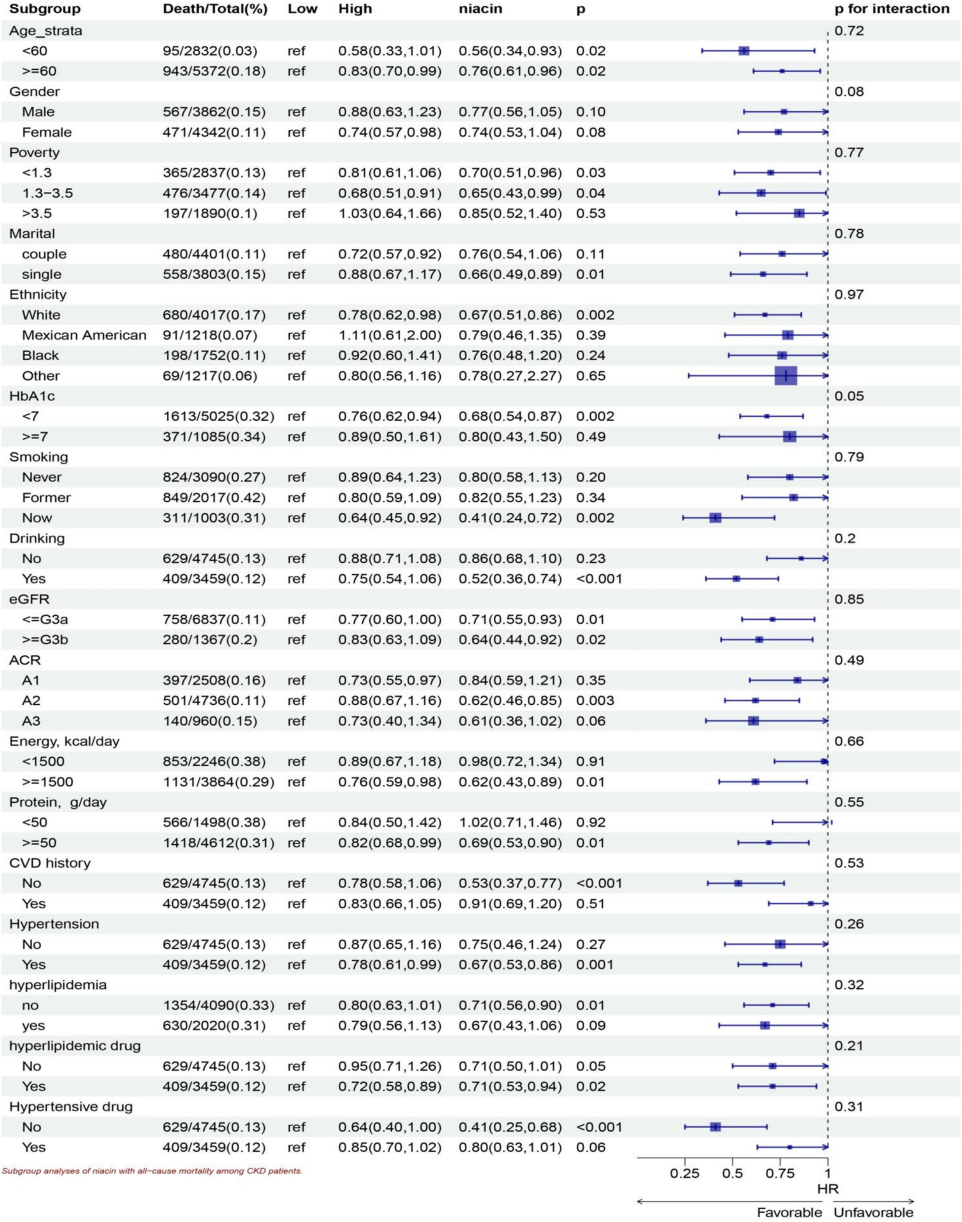
Subgroup analyses of niacin with all-cause mortality among CKD patients. HR (95% CI) was assessed by Cox proportional hazards model. The model was adjusted for consistent confounding factors, including age, gender, ethnicity, marital, poverty, education, smoking, drinking, obesity, HbA1c, Hb, NLR, uric acid, blood urea nitrogen, bicarbonate, creatinine, uACR (ln-transformed), albumin, hdl cholesterol, SII(ln-transformed), dietary energy kcal, dietary protein, dietary fiber intake, dietary sodium, dietary potassium, CVD history, hypertension, hyperlipidemia, the use of hyperlipidemic drug, the use of hypertensive drug uACR, A1, <30 mg/g; A2 30–300 mg/g; A3 > 300 mg/g; eGFR, <=G3a, eGFR > = 45 mL/(min • 1.73 m^2^), > = G3b, eGFR >45 mL/(min • 1.73 m^2^).

**Figure 6:**
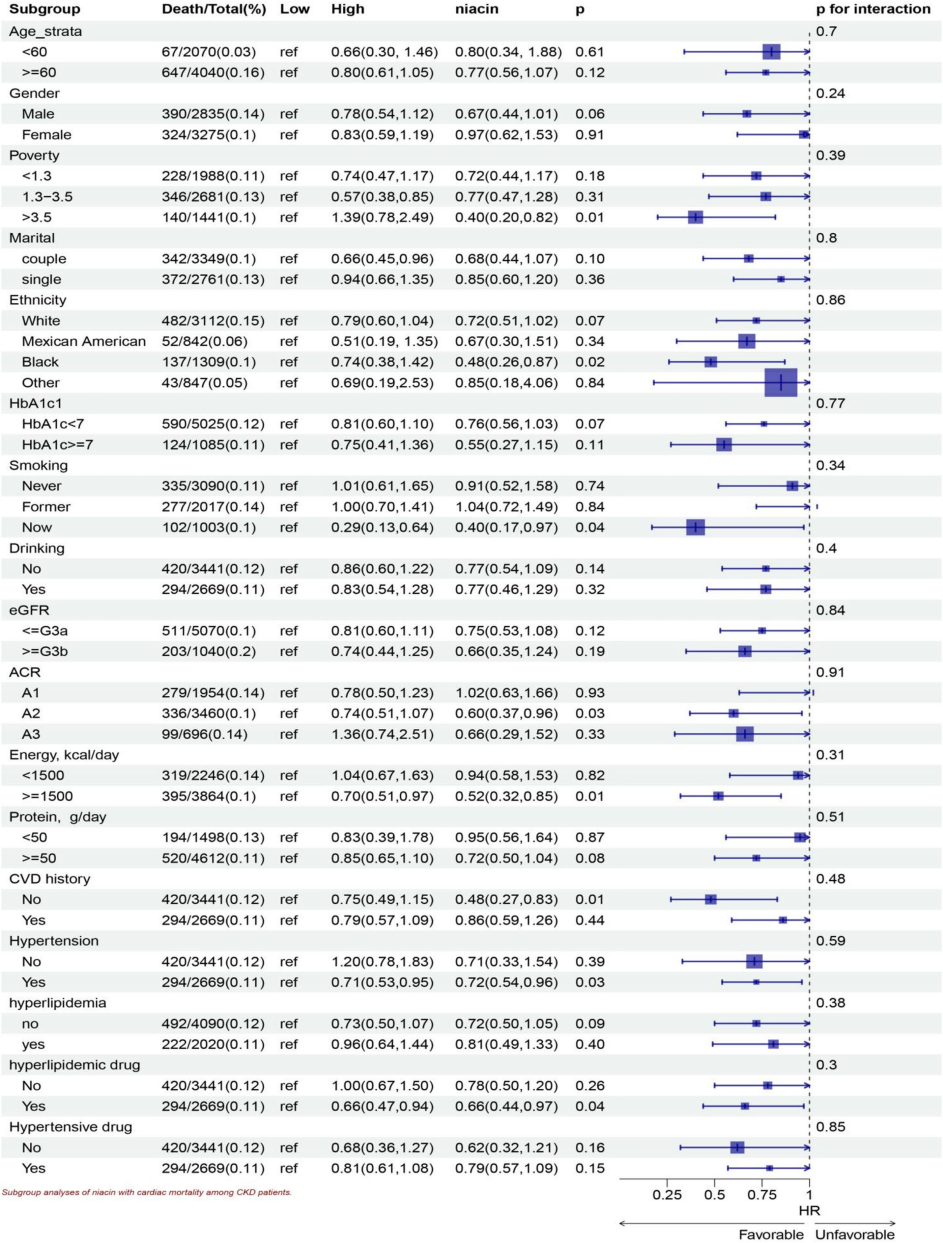
Subgroup analyses of niacin with CVD mortality among CKD patients. HR (95% CI) was assessed by Cox proportional hazards model. The model was adjusted for consistent confounding factors, including age, gender, ethnicity, marital, poverty, education, smoking, drinking, obesity, HbA1c, Hb, NLR, uric acid, blood urea nitrogen, bicarbonate, creatinine, uACR (ln-transformed), albumin, hdl cholesterol, SII(ln-transformed), dietary energy kcal, dietary protein, dietary fiber intake, dietary sodium, dietary potassium, CVD history, hypertension, hyperlipidemia, the use of hyperlipidemic drug, the use of hypertensive drug uACR, A1, <30 mg/g; A2 30–300 mg/g; A3 > 300 mg/g; eGFR, <=G3a, eGFR > = 45 mL/(min • 1.73 m^2^), > = G3b, eGFR >45 mL/(min • 1.73 m^2^).

Stratified analyses show significant association in all ages, the White, lower family income, smoking, drinking, HbA1c < 7, all eGFR strata, more energy intake, with and without hyperlipidemic medication, without hypertensive medication, more protein intake, hypertension, without CVD history, without hyperlipidemia subgroup for niacin intake in all-cause mortality (Figure 5). Stratified analyses show significant association smoking, more energy intake, hypertension, without CVD history, with hyperlipidemic medication, subgroup for niacin intake in CVD mortality (Figure 6).

## Discussion

Our study is the first large-scale prospective cohort study to explore the association between dietary niacin intake and all-cause and cardiovascular mortality in patients with CKD. Our results showed that higher dietary niacin intake was associated with lower all-cause mortality and cardiovascular mortality. The results are consistent after adjusting for multiple confounding factors. However, the relationship between dietary niacin intake and mortality in CKD patients remains unclear.

Dyslipidemia (16), characterized by low-density lipoprotein cholesterol (LDL-C), elevated triglycerides, and decreased HDL-C, is common in patients with CKD and significantly leads to cardiovascular mortality (17). Numerous studies have demonstrated that dyslipidemia exacerbates renal disease (18, 19) while lipid-lowering strategies have been shown to slow the progression of this condition in both humans (20, 21) and experimental animals (22–24). Niacin was first used as a therapeutic drug to reduce blood lipids (25). A post-hoc analysis of AIM-HIGH showed that extended-release niacin slowed the decrease in eGFR, improved triglyceride and high-density lipoprotein-cholesterol concentrations, and a small subgroup of subjects with baseline dyslipidemia showed possible benefit (26). Our results showed that in subgroup analysis, higher niacin intake has protective significance in patients with lipid-lowering drugs, and all-cause death and cardiovascular death have decreased 28, 34% respectively, but the protective effect in patients with dyslipidemia has not reached statistical significance. This may be related to the decrease in blood lipid after taking lipid-lowering drugs in patients with dyslipidemia. This finding is consistent and reasonable with the existing underlying theory.

Endothelial dysfunction is a hallmark of CKD and is a key factor in the development of atherosclerosis and cardiovascular complications (8, 27). Niacin improves endothelial function by increasing the bioavailability of nitric oxide (NO), thereby enhancing vasodilation and reducing vascular inflammation (9, 10). In our study, niacin was more effective in reducing mortality of patients with hypertension. For each unit of niacin intake, cardiovascular mortality is reduced by 28%, and all-cause mortality is reduced by 33%. Meanwhile, each unit of niacin intake reduced all-cause death by 59% in patients without hypertensive medication. Considering the protective effect of niacin, improved endothelial function can better regulate blood pressure and vascular tone, thereby reducing mortality in patients with CKD.

Niacin has the potent antioxidant and anti-inflammatory properties (28). Previous basic study show that niacin supplementation helps to attenuate histological injury and mitigate upregulation of oxidative and inflammatory systems in the remnant kidney of rat (29). At the same time, niacin can play a role in improving oxidative stress, inflammation, and endothelial dysfunction (29), thus reducing proteinuria and eGFR decline. In our study, the protective effect of niacin remained after adjusting for the inflammatory markers NLR and SII. After adjusting for blood lipids, the protective effect of niacin also remained, considering the anti-inflammatory effect of niacin independently of its lipid-lowering effect (30, 31).

Niacin has been reported to increase blood glucose levels in diabetic patients (32, 33). In our study, in the Hb1Ac <7 subgroup, the HR of higher dietary intake of niacin and all-cause and cardiovascular mortality were 0.76 (0.62, 0.94) and 0.81(0.6, 1.01) (p for interaction >0.05). In the Hb1Ac ≥ 7 subgroup, the protective effect of niacin did not reach statistical significance. Considering the effect of niacin on elevated glucose and the effect of diabetes itself on CKD patients, combined with the results of our subgroup analysis, we believe that increased niacin intake may be more strongly associated with a lower mortality risk in CKD patients with Hb1Ac <7.

Another notable finding of this study is that high energy intake patients benefit more from dietary niacin intake. In the high energy intake group, higher niacin intake can reduce all-cause mortality by 24% and CVD mortality by 30%, respectively. The synthesis of niacin requires energy, and high energy intake promotes the synthesis and utilization of niacin, and promotes cell function and energy balance (34). At the same time, adequate energy intake helps to maintain overall health, and niacin supplementation may provide additional protection. Niacin intake can reduce all-cause mortality in the higher protein intake group, with 18% decreased mortality. High protein intake may lead to increased nitrogen load, and the metabolic effect of niacin helps to improve nitrogen utilization and reduce renal burden (35). At the same time, sufficient protein and niacin combination can promote muscle synthesis and maintain the overall nutritional status, thus improving the survival rate.

The strength of our study is the use of a large sample size representing adults across the US to include the national population. Furthermore, our study spans 16 years and is the largest study cohort to date on the association between dietary niacin intake and CVD and all-cause mortality, filling a gap on the relationship between dietary niacin and CKD. Our study still has its limitations. First, since our study design is observational, a causal relationship cannot be established. Second, although dietary data were obtained from two 24 h dietary recalls, the presence of recall bias cannot be completely excluded, in which participants may inadvertently ignore the specific food items they consumed, resulting in an underestimation or overestimation of portion sizes. Furthermore, it is possible that some potential confounders are not adequately considered to allow the presence of residuals and unrecognized confounders that cannot be completely ruled out. Unmeasured or unknown factors could still influence the observed associations. Additionally, the inherent limitations of the observational study design should be acknowledged, as it precludes establishing causal relationships. While associations can be identified, causality cannot be definitively determined without randomized controlled trials.

In conclusion, our study showed that higher dietary niacin intake is associated with lower CKD all-cause mortality and cardiovascular mortality. Moreover, correlations still exist within the subgroups. The dose–response relationship between dietary nicotinate intake and reduced all-cause and CVD mortality in CKD patients requires further investigation to determine the optimal intake level. Future large-scale randomized trials are needed to further confirm the role of niacin in improving long-term outcomes in CKD patients.

## Data Availability

Publicly available datasets were analyzed in this study. This data can be found here: the NHANES dataset is publicly accessible via the NCHS, a division of the CDC. You can find the dataset at this website: https://www.cdc.gov/nchs/nhanes/.

## Glossary

CKD: chronic kidney disease
CVD: cardiovascular disease
NHANES: National Health and Nutrition Examination Survey
RCS: restricted cubic splines
NCHS: National Center for Health Statistics
uACR: urine albumin-to-creatinine ratio
eGFR: estimated glomerular filtration rate
CKD-EPI: Chronic Kidney Disease Epidemiology Collaboration
MEC: Mobile Inspection Center
USDA: United States Department of Agriculture
FNDDS: Food and Nutrient Database for Dietary Studies
BMI: body mass index
BP: blood pressure
NDI: National Death Index
ICD: International Classification of Diseases
HDL: high-density lipoprotein
LDL: low-density lipoprotein cholesterol
Hb: hemoglobin
HRs: hazard ratios
CIs: confidence intervals
CDC: Center for Disease Control and Prevention
SBP: systolic blood pressure
DBP: diastolic blood pressure
NADP: nicotinamide adenine dinucleotide phosphate
NAD: nicotinamide adenine dinucleotide
AMPM: automated multiple-pass method
ICD-10: International Statistical Classification of Diseases
IQRs: interquartile ranges
SII: systemic immune inflammation Index
NLR: Neutrophil to lymphocyte count ratio
LDL-C: low-density lipoprotein cholesterol
HDL-C: high-density lipoprotein cholesterol
